# Implementing a modified World Health Organization safe childbirth checklist in health centers of Ethiopia: a pre and post intervention study

**DOI:** 10.1186/s12884-021-03565-3

**Published:** 2021-01-22

**Authors:** Hailemariam Segni Abawollo, Zergu Tafesse Tsegaye, Binyam Fekadu Desta, Tsega Teferi Mamo, Haregewoin Getachew Mamo, Zebyderu Tesfay Mehari, Zenawork Kassa Gebremedhin, Ismael Ali Beshir

**Affiliations:** JSI/Transform: Primary Health Care Activity, Addis Ababa, Ethiopia

**Keywords:** WHO safe childbirth checklist, Essential childbirth supplies, Essential childbirth practices, USAID transform: primary health care

## Abstract

**Background:**

Childbirth is a complex process, and checklists are useful tools to remember steps of such complex processes*.* The World Health Organization safe childbirth checklist is a tool used to improve the quality of care provided to women giving birth. The checklist was modified by Ministry of Health and was introduced to health centers in Ethiopia by the USAID Transform: Primary Health Care Activity.

**Methods:**

A pre and post intervention study design with prospective data collection was employed. The availability of essential childbirth supplies and adherence of health care providers to essential birth practices were compared for the pre and post intervention periods.

**Results:**

The pre and post intervention assessments were conducted in 247 and 187 health centers respectively. A statistically significant improvement from 63.6% pre intervention to 83.5% post intervention was observed in the availability of essential childbirth supplies, t (389.7) = − 7.1, *p* = 0.000. Improvements in adherence of health care providers to essential birth practices were observed with the highest being at pause point three (26.2%, t (306.3) = − 10.6, *p* = 0.000) followed by pause point four (21.1%, t (282.5) = − 8.0, *p* = 0.000), and pause point two (18.2%, t (310.8) = − 9.7, p = 0.000). The least and statistically non-significant improvement was observed at pause point one (3.3%, t (432.0) = − 1.5, *p* = 0.131).

**Conclusion:**

Improvement in availability of essential childbirth supplies and adherence of health care providers towards essential birth practices was observed after introduction of a modified World Health Organization safe childbirth checklist. Scale up of the use of the checklist is recommended.

**Supplementary Information:**

The online version contains supplementary material available at 10.1186/s12884-021-03565-3.

## Background

Globally, maternal mortality is unacceptably high with the majority of the deaths being potentially preventable and occurring in low- and middle-income countries. Around 830 women die daily from pregnancy and childbirth related complications [1].

Since 1990 many sub-Saharan African countries have been successful in reducing their rates of maternal mortality. Sustainable Development Goal 3 includes a target that aims to reduce the global maternal mortality ratio to less than 70 per 100,000 live births, with no country having a maternal mortality rate of more than twice the global average [2, 3].

Around 75% of all maternal deaths are due to severe bleeding, infections, high blood pressure during pregnancy, complications from delivery, and unsafe abortions [4].

Maternal and newborn health are closely linked. Approximately 2.7 million newborn babies died in 2015, and an additional 2.6 million were stillborn. It is of paramount importance that all births are attended by skilled health professionals, as timely management can make the difference in the lives of both the mother and the baby [5, 6].

Childbirth is a complex process, and it is essential to remember to provide everything that is needed to ensure both the mother and newborn receive the safest care possible. Checklists are essential tools that organize such complex and important processes [7, 8]*.* The World Health Organization (WHO) safe childbirth checklist (SCC) is one of these tools, used to improve the quality of care provided to women during childbirth and in the hours afterwards. It is a well-organized list of evidence-based essential birth practices (EBPs) which focus on top causes of maternal deaths, intrapartum-related stillbirths, and early neonatal deaths [9].

In Namibia, the use of the WHO SCC showed an improvement in average EBPs delivered from 68 to 95% [10]. In Rajasthan, India, the use of the WHO SCC increased providers’ performance of best practices, reflecting improvements in quality of facility childbirth care for women and newborns [11]. In Uttar Pradesh, India, birth attendants’ adherence to EBPs was higher in facilities that used the coaching-based WHO SCC program than in those that did not [12]. In Aceh, Indonesia, use of the WHO SCC improved the quality of maternal care and overall birth experiences [13].

The WHO SCC was modified by Ministry of Health in Ethiopia and the USAID Transform: Primary Health Care Activity has introduced it to its catchment health centers and has carried out pre and post intervention assessment of changes.

## Methods

### Aim

The aim of this study was to assess pre and post intervention changes in availability of essential childbirth supplies and adherence of health care providers to EBPs.

### Design

A health facility-based pre and post intervention study design with prospective data collection was employed to assess the changes pre and post the intervention.

### Setting

The assessment was conducted in health centers within four regions of Ethiopia (Amhara, Oromia, Tigray, and South Nations Nationalities and Peoples’) where USAID Transform: Primary Health Care Activity has been operating since January 2017. The Activity has divided the four regions into 29 clusters (10 in Oromia, 9 in Amhara, 6 in South Nations Nationalities and Peoples’, and 4 in Tigray) which are constituted of zones and woredas with their primary hospitals, health centers and health posts. Health centers are a part of the primary health care of a three-tier health service delivery system of the country and serve as referral centers for cases from health posts and homes. Health centers provide basic emergency obstetric and newborn care (BEmONC) services and their maternity units are mainly run by midwives.

### Intervention

One cluster per region was selected purposively as utilization of WHO SCC had not yet started at health centers of the selected clusters. A similar structured assessment tool was used for both pre and post intervention assessments where data on availability of essential childbirth supplies and adherence of health care providers to EBPs were collected.

In the modified checklist, some items of the original WHO SCC were removed while some were added, (Table 1). The original WHO SCC and the modified WHO SCC are found as supplementary files.

**Table 1:** List of items removed and added to the original WHO SCC in the development of the modified, Ethiopian version checklist

Pause points (PP)	Items removed	Items added
PP-1	–	“Quick check performed?” “Antiretroviral medicine?”
PP-2	–	“Antiretroviral medicine?”
PP-3	“Is mother bleeding abnormally?” “Does the mother need to start, ✓ Antibiotics? ✓ Magnesium sulfate and antihypertensive?”	Components of essential newborn care. List of both maternal and newborn danger signs.
PP-4	“Confirm stay at facility for 24 h after delivery” “Is mother’s blood pressure normal?” “Is baby feeding well?”	“Refer mother to three postnatal visits (6–24 h, 3 days, 7 days) and an immunization visit at 6 weeks.”

**Fig. 1:**
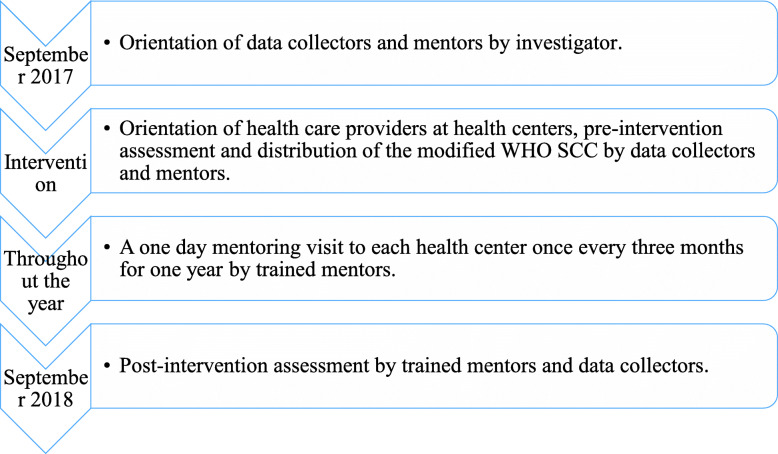
Sequential events of the implementation of the modified WHO SCC at USAID Transform: Primary Health Care Activity’s health centers, Ethiopia

Based on the WHO SCC implementation guide, in September 2017, an orientation on the modified WHO SCC was conducted for data collectors and mentors (one regional officer per region and three to five cluster officers per cluster, who are master of public health degree holders with midwifery, nursing or public health officer backgrounds) and print outs of the checklist were distributed to the clusters. The cluster staff then conducted onsite orientations to health care providers (midwives and clinical nurses who had undergone two to four years of training), distributed the checklists, and collected pre intervention assessment data. The pre intervention data were collected by interviewing one health care provider per facility and directly observing the facility for presence of essential childbirth supplies. Regular, one-day mentoring visits were carried out every three months where all technical staff were mentored. The mentors used orientation materials prepared for the purpose (from the WHO SCC implementation guide), the WHO SCC implementation guide, and copies of the checklist to practice, discuss and fix technical and supply related gaps (if any). The post intervention assessment was conducted a year later using the same assessment tool and the same way of data collection as the one used at the pre intervention stage. Adherence to practices was assessed through interviews of providers by asking whether they carried out the EBPs mentioned in the modified WHO SCC or not, but a completed checklist was not considered as adherence to practice. Client records were reviewed to check for consistent and correct use of the checklist. (Fig. 1).

Data were cleaned, edited and entered to a data entry template with analysis being conducted using SPSS version 25. The availability of essential supplies and adherence of health care providers to essential birth practices were compared during the pre and post intervention stages using independent samples t-test and Levene’s test, with the level of significance being determined at a *p* < 0.05.

### Ethical considerations

This assessment was conducted as part of USAID Transform: Primary Health Care Activity’s interventions which is being implemented in Ethiopia under a cooperative agreement number of AID-663-A-17-00002.

## Results

Data were collected from 247 and 187 health centers during the pre and post intervention periods, respectively.

Levene’s test for equality of variances assessment revealed that homogeneity of variances was violated for all the variables except for pause point one, quick checks for danger signs performed before referral or admission of mothers, partograph use for all laboring mothers at the facility, and HIV testing and treatment services for both the mother and baby during antenatal care (ANC). Therefore, a Welch t-test was run to determine if there were differences in adherence of health care providers to EBPs between pre and post interventions and an independent-samples t-test was run for variables that met the homogeneity assumptions.

A statistically significant improvement from a pre intervention score of 63.6 to 83.5% post-intervention was observed in the availability of essential childbirth supplies in selected health centers of Ethiopia one year after the introduction of the modified WHO SCC, t (389.7) = − 7.1, *p* = 0.000, (Table 2).

**Table 2:** Pre and post modified WHO SCC intervention changes on the availability of essential childbirth supplies at selected health centers supported by USAID Transform: Primary Health Care Activity, Ethiopia

	Before			After			95% CI for Mean Difference	t	df	
	M	SD	n	M	SD	N				P
Availability of necessary supplies in labor, delivery and postnatal rooms	**63.6**	**33.0**	**247**	**83.5**	**22.5**	**150**	**−25.4, − 14.4**	−7.1^*^	**389.7**	**0.000**
Oxytocin	78.5	41.1	247	96.2	9.2	187	− 23, −12.3	−6.5^*^	278.3	0.000
Intravenous fluids	76.1	42.7	247	96.6	6.3	187	−25.9, −15.0	−7.4^*^	260.2	0.000
Antibiotics	65.6	47.6	247	91.9	11.0	187	−32.5, −20.1	− 8.4^*^	280.2	0.000
Magnesium sulfate	67.6	46.9	247	93.1	9.9	187	−31.6, −19.5	−8.3^*^	274.4	0.000
Antiretroviral drugs	50.6	50.1	247	80.1	19.4	187	− 36.3, − 22.6	−8.4^*^	335.6	0.000
Vitamin K	34.4	47.6	247	85.2	20.9	187	− 57.4, − 44.1	15.0^*^	356.2	0.000
Tetracycline eye ointment	59.1	49.3	247	88.9	13.7	187	−36.3, −23.3	−9.1^*^	295.0	0.000
Bacillus Calmette-Guerin vaccine	57.1	49.6	247	90.2	13.7	187	− 39.7, − 26.6	10.0^*^	294.4	0.000
Oral Polio Vaccine	72.5	44.8	247	94.4	8.3	187	− 27.7, − 16.2	−7.5^*^	268.4	0.000
Gloves	74.9	43.4	247	95.5	7.2	187	−26.1, −15.0	−7.3^*^	263.9	0.000
Syringes	77.3	42.0	247	95.7	6.8	187	−23.7, −13.0	−6.8^*^	263.0	0.000
Soap	63.6	48.2	247	92.3	11.2	187	−35, −22.5	−9.0^*^	280.6	0.000
Water	58.7	49.3	247	90.9	12.8	187	−38.6, −25.8	−9.8^*^	288.6	0.000
Alcohol hand rub	54.7	49.9	247	87.6	16.1	187	−39.6, −26.3	−9.7^*^	310.7	0.000

* *p* < .05, *M* mean, *SD* standard deviation, *CI* Confidence interval, *df* degree of freedom, *n* preintervention sample size, *N* postintervention sample size

A statistically significant improvement in the adherence of health care providers to EBPs was observed post intervention which was one year after the introduction of the modified WHO SCC. The highest level of improvement was observed at pause point three (26.2%, t (306.3) = − 10.6, *p* = 0.000) followed by pause point four (21.1%, t (282.5) = − 8.0, p = 0.000) and pause point two (18.2%, t (310.8) = − 9.7, p = 0.000). The least and statistically non-significant improvement was observed at pause point one (3.3%, t (432.0) = − 1.5, *p* = 0.131). (Table 3).

**Table 3:** Pre and post modified WHO SCC intervention adherence of health care providers to essential birth practices at selected health centers supported by USAID Transform: Primary Health Care Activity, Ethiopia

	Before			After			95% CI for Mean Difference	t	df	
	M	SD	n	M	SD	N				p
**Pause point 1**	**70.8**	**22.8**	**247**	**74.1**	**23.4**	**187**	**− 7.8, 1.0**	**−1.5**	**432.0**	**0.131**
Quick checks for danger signs performed before referral/admission of mothers	75.2	42.8	247	73.3	43.0	187	− 6.3, 10	0.4	432.0	0.658
Functional referral linkages and feedback mechanisms for both mother and newborn	39.2	47.8	247	51.2	49.6	187	−21.3, −2.7	−2.5^*^	392.7	0.012
Partograph being used for all laboring mothers at the facility	81.3	38.1	247	82.5	37.1	187	−8.3, 6.0	−0.3	432.0	0.753
HIV testing and treatment services for the mother and baby both during antenatal care and delivery	87.4	33.2	247	89.6	30.3	187	−8.3, 3.9	−0.7	432.0	0.472
**Pause point 2**	**71.2**	**27.5**	**247**	**89.4**	**8.9**	**187**	**−21.9, −14.5**	−9.7^*^	**310.8**	**0.000**
Relatives are encouraged to accompany laboring mothers during labor and delivery	92.5	26.0	247	98.1	12.6	187	−9.3, −1.8	−2.9^*^	374.8	0.004
Essential supplies for the mother kept at bed side before delivery	63.0	44.9	247	85.9	12.6	187	−28.8, −17.0	−7.6^*^	295.4	0.000
Essential supplies for the baby kept at bed side before delivery	58.0	40.3	247	84.1	12.6	187	−31.5, −20.8	−9.6^*^	306.8	0.000
**Pause point 3**	**61.2**	**36.7**	**247**	**87.4**	**11.4**	**187**	**−31.1, −21.3**	−10.6^*^	**306.3**	**0.000**
Placing baby in skin to skin contact	71.1	45.3	247	92.3	9.8	187	−27.1, −15.4	−7.1^*^	275.7	0.000
Breast feeding initiated within one hour of birth if mother and child are well	71.6	45.1	247	92.7	9.3	187	−26.9, −15.3	−7.2^*^	273.1	0.000
Vitamin K given 1 mg intramuscular on anterior mid-thigh	43.1	49.5	247	77.8	21.0	187	− 41.5, −27.7	−9.9^*^	350.7	0.000
Tetracycline eye ointment given in both eyes	57.0	49.5	247	87.2	14.9	187	−36.8, −23.6	−9.1^*^	302.5	0.000
Baby weighted and recorded	67.5	46.8	247	89.8	11.5	187	−28.4, −16.2	−7.2^*^	284.1	0.000
Bacillus Calmette-Guerin and oral polio vaccines given before discharge	57.0	49.5	247	84.7	15.5	187	− 34.4, −21.2	−8.3^*^	307.3	0.000
**Pause point 4**	**69.9**	**40.2**	**247**	**91.0**	**9.6**	**187**	**−26.3, −15.9**	−8.0^*^	**282.5**	**0.000**
Counselled on and offered family planning	71.8	44.9	247	92.3	9.4	187	−26.2, −14.7	−7.0^*^	273.9	0.000
Exclusive breast feeding for 6 months	75.5	42.9	247	93.2	8.4	187	−23.2, −12.2	− 6.3^*^	270.4	0.000
Immunization	69.4	46.0	247	91.7	10.3	187	−28.3, − 16.4	−7.4^*^	278.1	0.000
Hygiene	66.9	46.9	247	90.3	11.4	187	−29.5, − 17.3	−7.5^*^	283.5	0.000
Danger signs in both mother and newborn	71.0	45.2	247	91.3	9.9	187	−26.2, − 14.5	−6.8^*^	276.9	0.000
Need for postnatal care and follow up arranged	64.5	47.7	247	87.1	13.0	187	−28.9, −16.3	−7.1^*^	293.1	0.000

* p < .05, *M* mean, *SD* standard deviation, *CI* Confidence interval, *df* degree of freedom, *n* preintervention sample size, *N* postintervention sample size

## Discussion

In this pre and post intervention study, changes in the availability of essential childbirth supplies in labor, delivery and postnatal care units, and adherence of health care providers to EBPs (which are known to have a high impact on reducing preventable maternal and neonatal deaths around the time of delivery) were assessed a year after the introduction of a modified version of the WHO SCC.

Statistically significant changes were observed in the availability of essential childbirth supplies in labor, delivery and postnatal care units a year after the introduction of the modified WHO SCC. The changes in the availability of essential childbirth supplies observed in this study are similar with the findings of another study conducted in Uttar Pradesh, India which is a comparable setup with where this study was conducted [14, 15].

The magnitude of increment in adherence of health care providers to EBPs from the pre to post intervention was found to be similar with findings from studies at other similar settings of the world. A cluster randomized controlled trial in Uttar Pradesh, India, showed a similar improvement in adherence of health care providers to EBPs in intervention groups as compared to control [12]. A study at Gobabis district hospital, Namibia, also showed an improvement of average EBPs delivered from 68 to 95% which is comparable with this study [10]. Similar improvements in adherence of health care providers to EBPs were reported in other studies conducted in Aceh, Indonesia, and Rajasthan, India [11, 13].

In contrast to this study, the highest increase in EBPs was at PP-4 in a Rwandan study which may be due to difference in the evaluation period where the Rwandan post intervention assessment was carried out three weeks after SCC introduction while this current study’s post intervention assessment was carried out a year after introduction of the SCC [16].

Experience with a context-specific modified WHO SCC at two tertiary care settings in Sri Lanka showed that the mean adherence to the checklist practice was 52.7 and 32.2%, both of which are lower than this study’s findings. The difference in mean adherence can be explained by the difference in the settings where the studies were conducted. The Sri Lankan study used tertiary care centers where lack of staff numbers, lack of enthusiasm, inadequate training and advice on use of the checklist and lack of supervision from Ministry/institution levels were mentioned as reasons for low adherence. In contrast, this current study was conducted in primary care facilities where the health centers are not overwhelmed with cases and health care providers have adequate time to consistently and correctly use the checklist to guide their practices [17].

The average level of adherence of health care providers to checklist practices at another tertiary care center in Sri Lanka was found to be 71.3% which is comparable with the mean adherence at pause point one of this study. In addition to setting differences, increased workload, poor enthusiasm of health workers towards add on tasks to their routine schedules and level of user-friendliness of the checklist were mentioned in the Sri Lankan study as possible the reasons for the lower levels of adherence as compared to adherence at the other pause points of the current study [18].

Experience from 60 public health facilities in Uttar Pradesh, India, where improving adherence to EBPs using WHO SCC through peer coaching was conducted showed that 35 out of 39 (89.7%) EBPs had achieved > 90% adherence in the presence of a coach by the final month of the eight month long intervention, as compared with only 7 out of 39 (18%) practices during the first month. Despite the Hawthorne effect, the improvement in adherence to EBPs in the Indian study is comparable with the current study [19].

A research which aimed at testing a modified version of the WHO SCC in an Italian hospital showed that compliance to the checklist was high for midwives (96%), but very low for obstetricians (3%). The compliance of midwives in the Italian study is comparable with that of this current study but since there are no obstetricians working at health centers in Ethiopia a comparison cannot be made for that aspect of the study [20].

### Limitations

The findings of this study should be interpreted with consideration to some of its limitations which are:

- The post intervention assessment was conducted on a lower number of health centers (187) as compared to the pre intervention facilities (247) due to inaccessibility of some of the health centers following security related issues at the time of data collection.
- The interviews might have not been conducted with same interviewees during the pre and post intervention assessments due to the high turnover rates of health care providers.
- Interviews with health care providers were used to assess adherence to essential birth practices with no direct observation of actual practices and it is possible that the checklists were simply filled out after delivery or at discharge and not in real time.
- As the study was conducted at primary care facilities where only mid-level providers (midwives, nurses, and public health officers) work, findings of the study may not be generalized for secondary and tertiary care facilities where a mix of various cadres of health work.

## Conclusion

Improvements in availability of essential childbirth supplies at labor, delivery and postnatal care units and adherence of health care providers towards essential childbirth practices were observed a year after the introduction of a modified version of World Health Organization safe childbirth checklist at health centers of Ethiopia.

## Recommendations

Scale up of the use of the modified World Health Organization safe childbirth checklist at all health facilities in the country is recommended. A local study on the contribution of the modified World Health Organization safe childbirth checklist on maternal and perinatal outcomes is also recommended.

## Supplementary Information

**Additional file 1.**

## Data Availability

The datasets during and/or analyzed during this study are available from the corresponding author upon reasonable request.

## Abbreviations

AIDS: Acquired Immunodeficiency Syndrome
ANC: Antenatal Care
BEmONC: Basic Emergency Obstetric and Newborn Care
EBP: Essential Birth Practices
HIV: Human Immune Virus
PP: Pause Point
SCC: Safe Childbirth Checklist
SPSS: Statistical Package for Social Science Students
USAID: United States Agency for International Development
WHO: World Health Organization
